# Low Dose Nicotine Attenuates Aβ Neurotoxicity through Activation Early Growth Response Gene 1 Pathway

**DOI:** 10.1371/journal.pone.0120267

**Published:** 2015-03-27

**Authors:** Maoqiang Xue, Liuwei Zhu, Jie Zhang, Jinhua Qiu, Guicheng Du, Zhiliang Qiao, Guanghui Jin, Fengguang Gao, Qiqing Zhang

**Affiliations:** 1 College of Chemistry and Chemical Engineering, Xiamen University, Xiamen, Fujian, 361005, P.R.China; 2 Institute of Biomedical Engineering, Department of Biomaterials, College of Materials, Xiamen University, Xiamen, Fujian, 361005, P.R.China; 3 Department of Basic Medical Science, Medical College, Xiamen University, Xiamen, Fujian, 361005, P.R.China; 4 Institute of Biomedical Engineering, Chinese Academy of Medical Science & Peking Union Medical College, The Key Laboratory of Biomedical Material of Tianjin, Tianjin, 300192, P.R.China; Indiana School of Medicine, UNITED STATES

## Abstract

Epidemiological studies indicate that smoking is negatively correlated with the incidence and development of Alzheimer's disease (AD). Nicotine was reported to be the active factor. However, the detailed mechanisms still remain to be fully elucidated. Early growth response gene 1 (EGR-1) plays important roles in several important biological processes such as promoting cell growth, differentiation, anti oxidative stress, and apoptosis, but few in the pathogenesis of AD. In the present study, we show that nicotine can activate the MAPK/ERK/EGR-1 signaling pathway partially through α7 nAChR. In addition, the up-regulation of EGR-1 by nicotine can also increase the phosphorylation of CyclinD1 which contributes to the attenuation of amyloid-β (Aβ_25–35_) -induced neurotoxicity. Although nicotine and Aβ_25–35_ can activate EGR-1, the expression of EGR-1 is down-regulated following treatment with nicotine and Aβ_25–35_. This study demonstrates that low dose nicotine attenuates Aβ_25–35_-induced neurotoxicity *in vitro* and *in vivo* through activating EGR-1 pathway.

## Introduction

Alzheimer's disease (AD), the most common form of dementia, is characterized by memory loss and cognitive defects [1–3]. Nevertheless, the impact mechanisms of learning and memory deficits in AD remain poorly understood. There are two hallmarks for AD: extracellular amyloid-β (Aβ) plaques and intracellular neurofibrillary tangles formed by hyperphosphorylated Tau. Many factors are attributed to the progression of AD, such as oxygen free radicals and metal ions homeostasis [4, 5].

Epidemiological studies have consistently shown that the incidence of AD is twice lower in smokers than in age-matched controls [6]. Nicotine exposure was reported to improve cognitive function and plays an important role in the prevention of impairment of memory and learning in AD [7, 8]. However, the detailed mechanism is not fully understood.

The zinc finger transcription factor EGR-1, which belongs to a group of early response genes, was independently discovered by several laboratories [9–11]. The *egr-1* gene can be induced by environmental factors such as ultraviolet (UV), ionizing radiation and injury, and cellular signals including growth factors, neurotransmitters, and cytokines as well as stress signals [12]. Thus, EGR-1 is involved in a variety of cellular processes including growth, differentiation, neurite outgrowth, wound healing and apoptosis. EGR-1 also can regulate neuroendocrine genes to benefit neuroplasticity and perform neuroprotective role to prevent memory loss [13, 14]. A previous study found that EGR-1 directly transactivated *bim* gene expression to mediate apoptosis of rat cerebellar granule neurons [15]. In addition, microtubules are destabilized by overexpression of *egr-1* in rat hippocampus neurons which promotes Tau phosphorylation [16]. EGR-1 is sufficient to promote axonal elongation induced by sAPPs [17]. EGR-1 transcription factor represents a potential mediator for neuroprotection induced by glatiramer acetate in AD [18].

In our study, we found that nicotine can activate EGR-1 through MAPK/ERK signaling pathway by the activation of α7 nAChRs to attenuate the neurotoxicity of Aβ_25–35_. The activation of EGR-1 by nicotine contributes to up-regulating the expression of cyclinD1 and its phosphorylation. Our immunohistochemistry results indicate that Aβ_25–35_ can increase the phosphorylation of ERK1/2, and this process can be blocked by nicotine administration. Last but not least, the protein level of EGR-1 was significantly decreased by nicotine treatment which was up-regulated by Aβ_25–35_. In this study, we elucidated the partial mechanisms that underlie the activation of EGR-1 by nicotine, and provide theoretical knowledge for retarding the proceeding of AD.

## Materials and Methods

### Antibodies and Chemical reagents

The antibodies against ERK1/2, Thr202/Tyr204 phospho-ERK1/2, cyclinD1, Thr286 phospho-cyclinD1 were from Cell Signaling Technology; Nicotinic acetylcholine receptor α7 was from Abcam; EGR-1, and β-actin were from Santa Cruz Biotechnology. U0126 was from Cell Signaling Technology; α-BTX, nicotine, and Aβ25–35 were from Sigma-Aldrich. Unless specified, all chemicals were obtained from Sigma-Aldrich Co (St. Louis, MO).

### Cell Culture

SH-SY5Y and rat PC12 pheochromocytoma cell lines were purchased from the Shanghai Institute of Material Medical, Chinese Academy of Science. SH-SY5Y cells were cultured in Dulbecco's modified Eagle's medium (Hyclone, Logan, UT, USA), and supplemented with 10% (v/v) fetal bovine serum (Hyclone) and 1×Penicillin-Streptomycin (100 U/ml or 100 mg/ml) (Invitrogen, Carlsbad CA, USA). PC12 cells were cultured in the same manner plus 5% (v/v) serum equinum.

### Animal model

Forty eight four-week-old male C57/BL/L6 mice were randomly divided into four groups, and all the animals are SPF (Specific Pathogen Free) laboratory animals and they are healthy, their weights ranged from 18g to 22g. The four groups included a control group, an AD model group, a standalone nicotine group and a nicotine-treatment AD model group. The standalone nicotine group and nicotine-treatment AD model group were injected 0.15 mg/kg nicotine using a subcutaneous injection every day for 2 weeks. Identical doses of sodium chloride were administered to the other two groups. Then the AD model group and nicotine-treatment AD model group were injected with 5 μL (5mg/ml) Aβ_25–35_ using a stereotaxis instrument (RWD, Shenzhen, China) and microinjector (KD Scientific, Holliston, MA, USA) into the left lateral ventricle of C57/BL/L6 mice (coordinates: anteroposterior, -2.0mm from the bregma; lateral, -2.0mm; dorsovental, -3mm), and other two groups were injected with identical doses of sodium chloride into the left lateral ventricle of C57/BL/L6 mice. After 16 days, the control group, the nicotine-treatment AD model group and the AD model group were practiced with the place navigation test and spatial probe test of water maze within 5 days. At the end of this period, all the mice were anesthetized with sodium pentobarbital (50μg/g) and euthanized by transcardial perfusion with ice-cold physiological saline. Brain cortex and hippocampus were collected for immunohistochemistry.

### Ethics statement

All animal procedures were in accordance with the National Institute of Health Guidelines for the Care and Use of Laboratory Animals and were approved by the Animal Ethics Committee of Xiamen University (IACUC #: XMULAC20130066).

### Plasmid and recombinant retrovirus construction

EGR-1 shRNA, the neuronal nicotinic acetylcholine receptors α7 shRNA plasmids and recombinant retroviruses were constructed. The sequences of EGR-1 and α7 shRNA were designed by Clontech RNAi designer (http://bioinfo.clontech.com), and were constructed into the retroviral vector RNAi-Ready pSIREN-RetroQ (Clontech). shRNA targets for hEGR-1: CGCCGAACACTGACATTTT; shRNA targets for hnAChRα7: AACAGTGCTGATGAGCGCTTTGAC. Recombinant retroviruses were packaged using GP2-293 cells according to the BD Retro-XTM Universal Packaging System protocol (BD Biosciences Clontech).

### MTT Assay for Cell Viability

To conduct MTT analysis, exponentially growing SH-SY5Y cells were plated into 96-well plates (5000 cells in 100 μL medium containing 10% fetal bovine serum per well). On the day following the plating of the cells, the culture medium was replaced with the serum free medium. The culture medium was prepared for each respective treatment, and culturing continued for another 48 to 72 hours. Cellular viability was measured in a 96-well plate by a quantitative colorimetric assay with MTT [19].

### Real-time Quantitative RT-PCR

Regular RT-PCR and quantitative RT-PCR (qRT-PCR) were performed as previously described [20]. For this assay, an ABI PRISM 7300 detection system was used with the primers as followings. The RT-PCR reactions were repeated at least three times.

β-actin (Forward: 5'-TCAGAAAGATTCCTACGTGGGCGA-3'; Reverse: 5'-TGTGGTGCCAGATCTTCTCCATGT-3'); EGR-1: primer1 (Forward: 5'-CCAAGGCCGAGATGCAATTGA-3'; Reverse: 5'-CCAGGGAGAAGCGGC CAGTAT-3'); EGR-1: primer2 (Forward: 5'-ATGCTTGCCCTGTCGAGTC-3'; Reverse: 5'-GGTATGCCTCTTGCGTTCATC-3'); nAChRα7 (Forward: 5'-AACAGTGCTGATGAGCGCTTTGAC-3'; Reverse: 5'-AGTGCTGCACATCAAAGGGAAACC-3').

### Western blot analysis

The Western blotting was carried out as described previously [20]. The antibodies and their dilutions were as following: Thr202/Tyr204 phospho-ERK1/2 (1:1000); Nicotinic acetylcholine receptor α7 (1:500); cyclinD1 (1:2000); Thr286 phospho-cyclinD1 (1:1000); EGR-1 (1:1500); β-actin (1:10000); ERK1/2 (1:2000). The intensities of immunoreactive bands were quantified by using an NIH Image tool.

### Cell apoptosis assay

Quantification of apoptosis by Annexin-V/PI staining was performed as described previously. Briefly, the cells were first collected after 24 hours drug treatment. Using a Beckman Coulter Elite flow cytometer (Beckman Coulter Electronics, FL, USA), 10,000 events per sample were collected into list mode files, and were analyzed by the WinMDI software.

### Immunohistochemistry and immunofluorescent staining

Immunohistochemistry and immunofluorescent staining was performed using an affinity-purified anti-EGR-1 and anti-pERK1/2 antibodies. The empirical method was identical as previously described [20]. After dewaxing, rehydration and washing, antigen retrieval was operated in EDTA buffer solution, and the endogenous peroxidase activity was inhibited by incubation with 3% H_2_O_2_ for 30 min. Then the sections were incubated with 5% normal fetal bovine serum at room temperature for 30 min. Then primary antibodies (EGR-1, 1:200; pERK1/2, 1:100) were loaded to brain slices (4°C, 12 h). After washing with PBS, peroxidase activity was developed using DAB as the chromogen (Maixin, Fuzhou, China).

The experimental results were photographed under a confocal microscope (×400). Nuclei were counterstained with DAPI, and the stained cells were analyzed and photographed under a confocal microscope(×400).

### Data and statistics analysis

All data were obtained from at least three different preparations. Statistical analysis of the data was performed with a two-tailed Student’s t-test using Graphpad Prism 5. Data are presented as the mean ± SEM. *p<0.05 was taken to represent statistical significance.

## Results

### Time course and concentration dependence of nicotine activation of EGR-1 in SH-SY5Y cells

**Fig 1:**
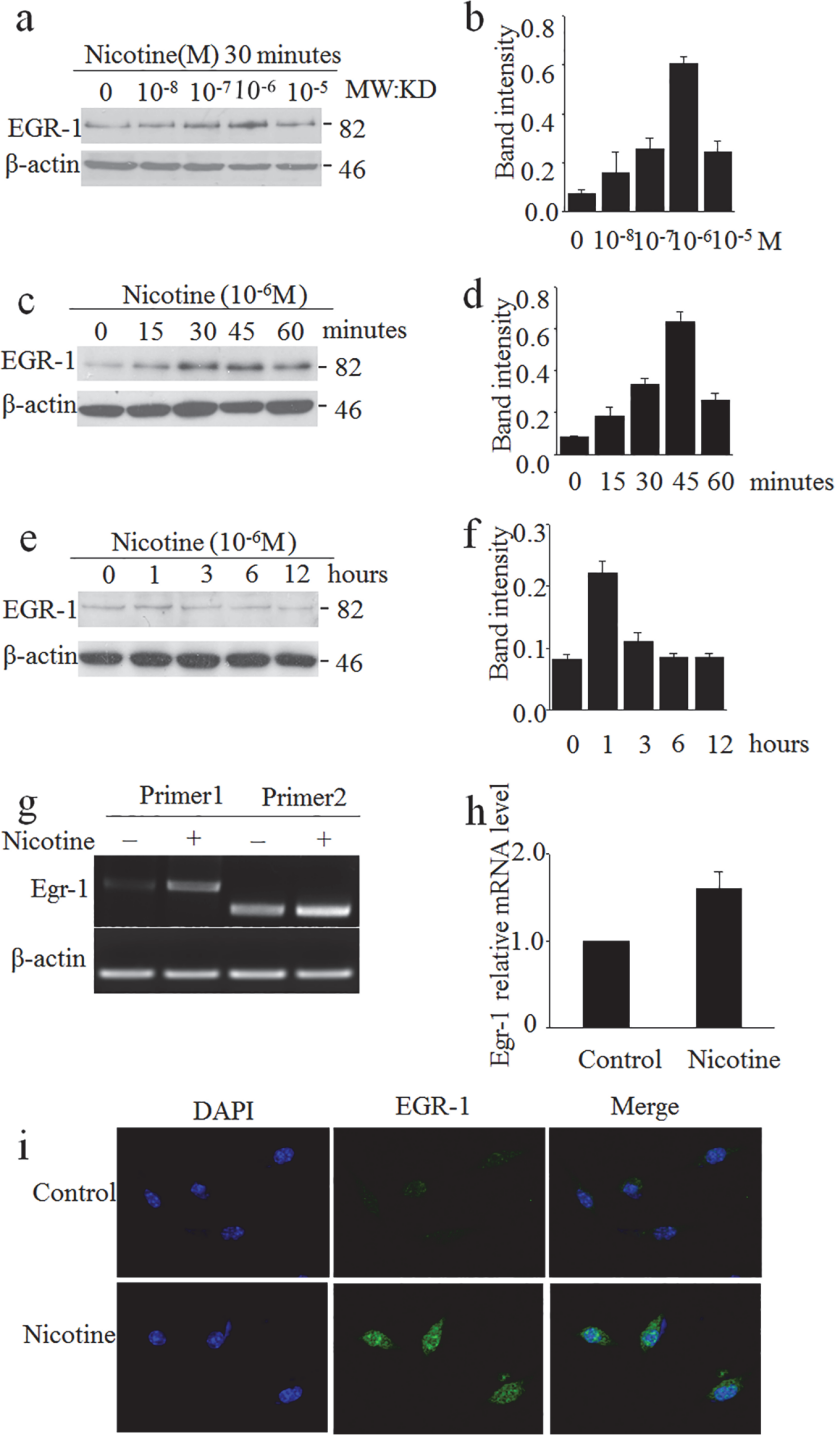
Time course and concentration dependence of nicotine activation of EGR-1 in SH-SY5Y cells. a, b) Nicotine activates EGR-1 in the nanomolar to micromole range when SH-SY5Y cells were treated with nicotine for 30 min; the relative level of EGR1 was quantified and presented in panel b. β-actin serves as control. c-e) The SH-SY5Y cells were with 10^-6^M nicotine by the indicated time, the relative level of EGR1 was quantified and presented in panel d and f respectively. β-actin serves as control. g) The mRNA level of EGR1 in SH-SY5Y cells after nicotine treatment was measured by RT-PCR. i) Immunofluorescence was shown when SH-SY5Y cells were treated by nicotine compared to control.

EGR-1 is an important transcription factor in AD [14–18, 21]. We detected the time course and concentration dependent effect of nicotine on the activation of EGR-1. As shown in Fig. 1, EGR-1 was dramatically activated by 10^–6^ M nicotine in 30 minutes (Fig. 1A). EGR-1 was quickly activated during time course, and the peak response occurred at 45 min (Fig. 1C, 1E). The activation of EGR-1 was then decreased with 1 hour treatment. This data confirms others' reports that nicotine could immediately activate EGR-1 and follow to basal level [22, 23]. The quantification data was presented on the right panels (Fig. 1B, 1D, 1F).

The mRNA level of EGR-1 was also investigated. Two pair primers were used. The EGR-1 mRNA was notably elevated when the cells were treated with 10^–6^ M nicotine for 30 min. The primer 1 of EGR-1 was used for the real-time PCR analysis. When treated with nicotine, the level of EGR-1 RNA was increased by roughly 1.5 times than the control (Fig. 1G). To confirm the western and RT-PCR data, endogenous EGR-1 was immunofluorescent stained in SH-SY5Y cell treated with nicotine. As shown in Fig. 1, the immunoactivity of EGR-1 was much higher after nicotine administration, especially in nucleus (Fig. 1H).

**Fig 2:**
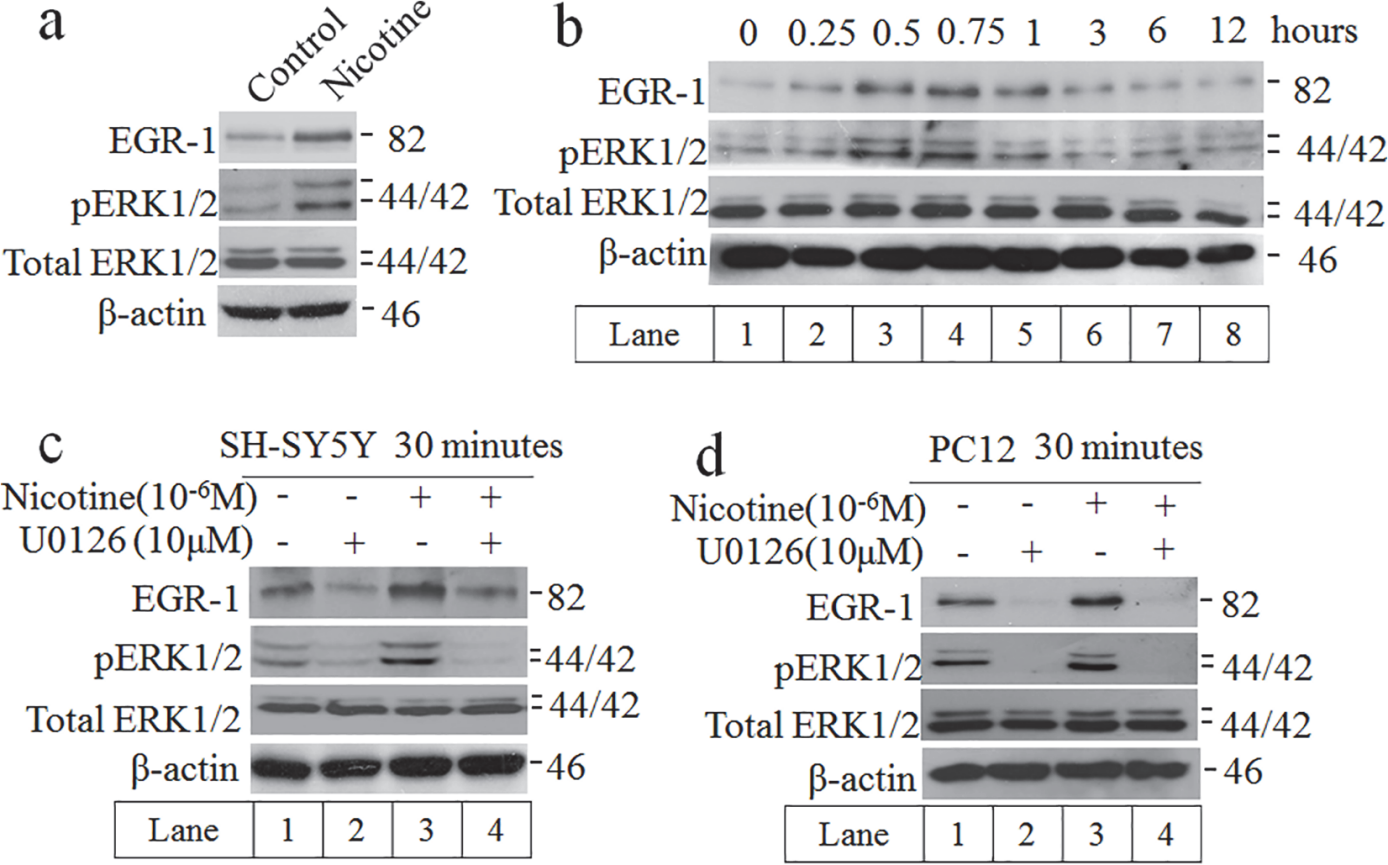
Nicotine activated EGR-1 through MAPK/ERK signaling pathway. a, b) Time-dependent induction of ERK1/2 phosphorylation by nicotine. When treated with nicotine, the changes in the protein level of EGR-1 and phosphorylated ERK1/2 were measured with Western blot. c, d) After treatment with nicotine (10^-6^M) and in the absence or presence of MEK1/2 inhibitor U0126 for 30 min, the EGR-1 and phosphorylated ERK1/2 levels were measured by Western blot in SH-SY5Y and PC12 cells.

Nicotinic activation of ERK1/2 regulates long-term memory and synaptic plasticity [21]. To analyze the activation of ERK1/2, the exponential SH-SY5Y cells were treated with 10^–6^ M nicotine for 30 min. Compared to the control, the phosphorylation of ERK1/2 was significantly increased while the total protein level remain unchanged (Fig. 2A). The experiment was then carried out for a longer time course (12 h) (Fig. 2B), and the result was similar to that of Fig. 2A. Additionally, the phosphorylated of ERK1/2 was maximal at 30 min for the nicotine group [24]. Notably, the peak response of ERK1/2 phosphorylation occurred prior to EGR-1, so it could be concluded that the activation of EGR-1 was correlated with ERK1/2 phosphorylation. Moreover, the MAPKs are important regulators of transcription [25]. To determine whether MEK-ERK1/2 pathway was involved in this process, the expression of EGR-1 protein in the presence and absence of the MEK1/2 inhibitor U0126 was measured. As shown in Fig. 2C, the induction of EGR-1 by nicotine was attenuated by U0126 which suggested that nicotine activates EGR-1 gene expression through ERK1/2 signaling pathway. Furthermore, similar results were also found in PC12 cells (Fig. 2D).

### Nicotine activation of MAPK/ERK pathway in SH-SY5Y cells is mediated by α7 nAchR

**Fig 3:**
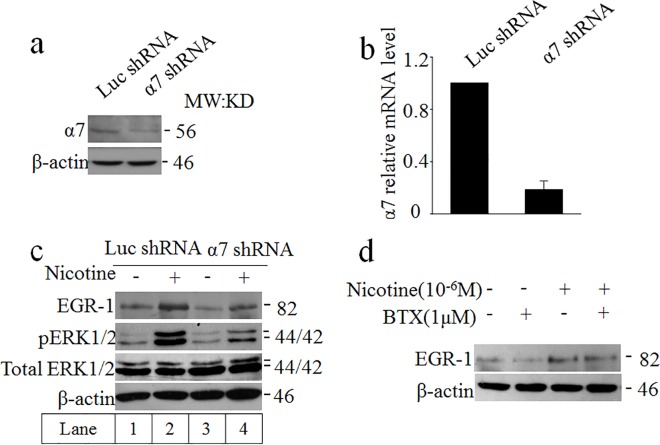
Nicotine activation of MAPK/ERK pathway in SH-SY5Y cells is mediated by α7 nAchR. We identified the effect of inhibition of α7 nAchRs expression by shRNA in Fig. 3. A, B) The effect of α7 shRNA by western blotting and real-time PCR. c) In the absence or presence of nicotine for 30 minutes, the EGR-1 and phosphorylated ERK1/2 levels were measured by western blot. d) When BTX, a specific α7 nAchR antagonist, was treated with nicotine, EGR-1 was detected.

It was known that nicotine and nicotinic agents possess neuroprotective effects, which are likely mediated by the stimulation of the α7 nicotinic receptors[26]. For instance, a prior study found evidence for a novel signaling route coupling the stimulation of α7 nAChR to the activation of ERK1/2 in a Ca^2+^ and PKA dependent manner [26]. Therefore, α7 nAchR shRNA was designed to silence α7 nAchR subunit. Western blotting and qRT-PCR analysis were performed to confirm the effective of nAchR shRNA (Fig. 3A and 3B). In Fig. 3C (refer to lane 1 and lane 2), nicotine activated EGR-1 and ERK1/2 phosphorylation; however, the administration of α7 nAchR attenuated the effect of nicotine. These findings suggested that the inhibition of α7 nAchRs expression by small hairpin RNA (shRNA) suppressed EGR-1 expression and ERK1/2 phosphorylation induced by nicotine. Stimulation of MAPK/ERK pathway with nicotine in cultured SH-SY5Y was also blocked by α7 nAchR antagonist α-bungarotoxin (BTX) (Fig. 3D). These results demonstrated that α7 nAchR function was necessary for nicotine-coupling to the MAPK/ERK cascade. It could be deduced that nicotine rapidly activates MAPK/ ERK1/2 in SH-SY5Y cells through α7 nAchR.

### Effect of nicotine on Aβ_25–35_-mediated cytotoxicity in SH-SY5Y cells

**Fig 4:**
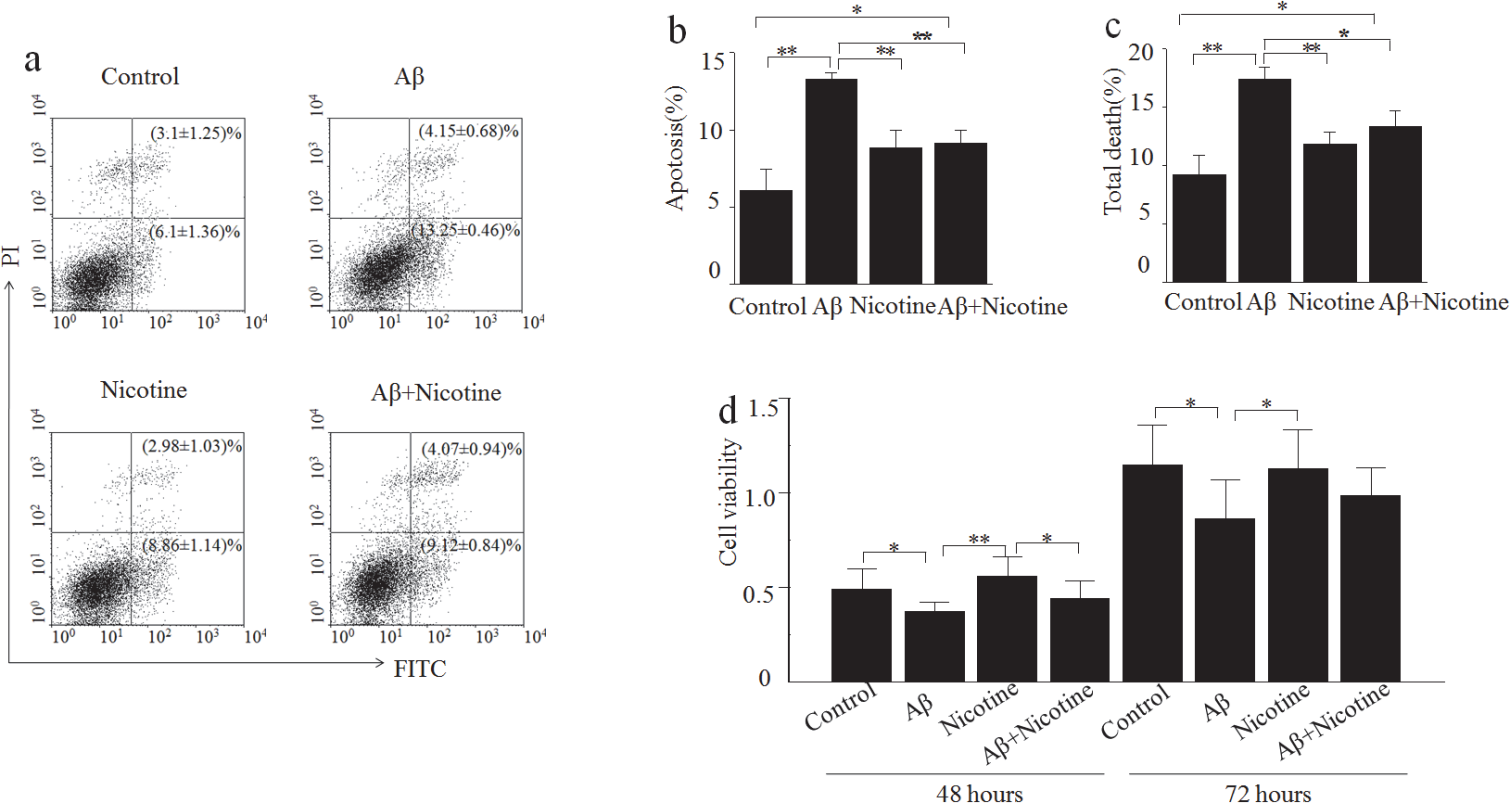
Nicotine attenuated Aβ_25–35_-induced neurotoxicity in human neuroblastoma SH-SY5Y cells. a-c) Apoptosis analysis of SH-SY5Y cell line treated with 10^–6^ M nicotine, 10^–6^ M Aβ_25–35_ or a combination of these drugs. Cells were exposed for 48 h. Double staining was used to distinguish between viable (lower left quadrant, annexinⅤ-negative,propidium iodide-negative), early apoptosis (lower right quadrant, annexinⅤ-positive, propidium iodide-negative), late apoptosis and necrotic (upper right quadrant, annexinⅤ-positive, propidium iodide-positive) and cell debris (upper left quadrant). Statistical analysis is shown in b. SH-SY5Y cells were exposed to 10^–6^ M nicotine, 10^–6^ M Aβ_25–35_ or a combination of these drugs for 48 h or 72 h, cell viability is measured by MTT, *P<0.05 verses control group.

Nicotine may cause an anti-apoptosis effect in its protection against salsolinol or Aβ-induced cytotoxicity in SH-SY5Y cells [27]. As shown in Fig. 4, the percentage of early and late apoptotic cells was significantly increased by 10^–6^ M Aβ_25–35_ treatment. The early apoptotic cells were 13.25% when treated with Aβ_25–35_ compared to the control of 6.1%. The neuronal toxicity of Aβ_25–35_ was attenuated when nicotine was added. The percentage of apoptotic cells decreased to 4.07%.

In SH-SY5Y cells, the MTT assay showed that Aβ_25–35_ induced cytotoxicity and significantly reduced cell viability at 1 μM compared to control cells (no Aβ_25–35_). The treatment of the cells with nicotine resulted in the reduction of Aβ_25–35_-induced cytotoxicity and significantly increased cell viability (Fig. 4C). The pretreatment of the cells with nicotine for 45 min or 2 hours also protected the cells from Aβ_25–35_-induced cytotoxicity. These results demonstrated that nicotine could protect cells from Aβ-induced cytotoxicity, implying that the treatment of the cells with nicotine protected against Aβ_25–35_-induced cell damage.

### EGR-1 regulates CyclinD1

**Fig 5:**
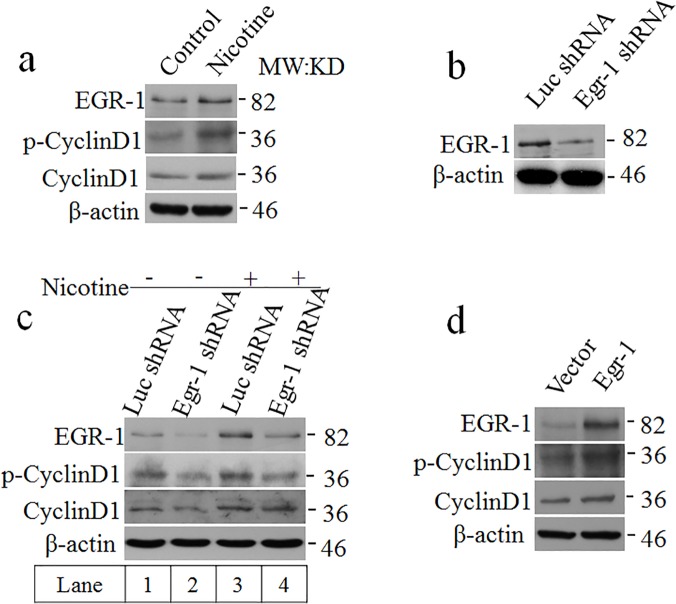
EGR-1 upregulates CyclinD1. a) SH-SY5Y cells were treated with nicotine for 45 min. CyclinD1 and its phosphorylation were detected by Western blotting. b) In SH-SY5Y cell line, the EGR-1 was blocked. c) Lane 3 and lane 4 are different from lane 1 and lane 2, which were treated with 10^–6^ M nicotine for 45 min. d) Following the overexpression of EGR-1 in SH-SY5Y cell line, the cyclinD1 and its phosphorylation were detected.

The above data suggested that EGR-1 be in vital for the neuroprotective effect of nicotine in Aβ-induced cytotoxicity in SH-SY5Y. Since EGR-1 is an important transcriptional factor, we then wondered which EGR-1 downstream gene was involved. CyclinD1 drew our attention. When SH-SY5Y cell line was treated by nicotine, the CyclinD1 was up-regulated (Fig. 5A). In the absent of EGR-1 by shRNA knockdown, the up-regulation of CyclinD1 by nicotine was reduced (Fig. 5B and 5C). With extra EGR-1 overexpression, CyclinD1 and its phosphorylation were significantly increased (Fig. 5D). It was hypothesized that EGR-1 might regulate CyclinD1 against Aβ_25–35_-induced cytotoxicity.

### Nicotine can be in antagonism to Aβ-induced EGR-1 and ERK1/2 activation in hippocampus in *vivo*

**Fig 6:**
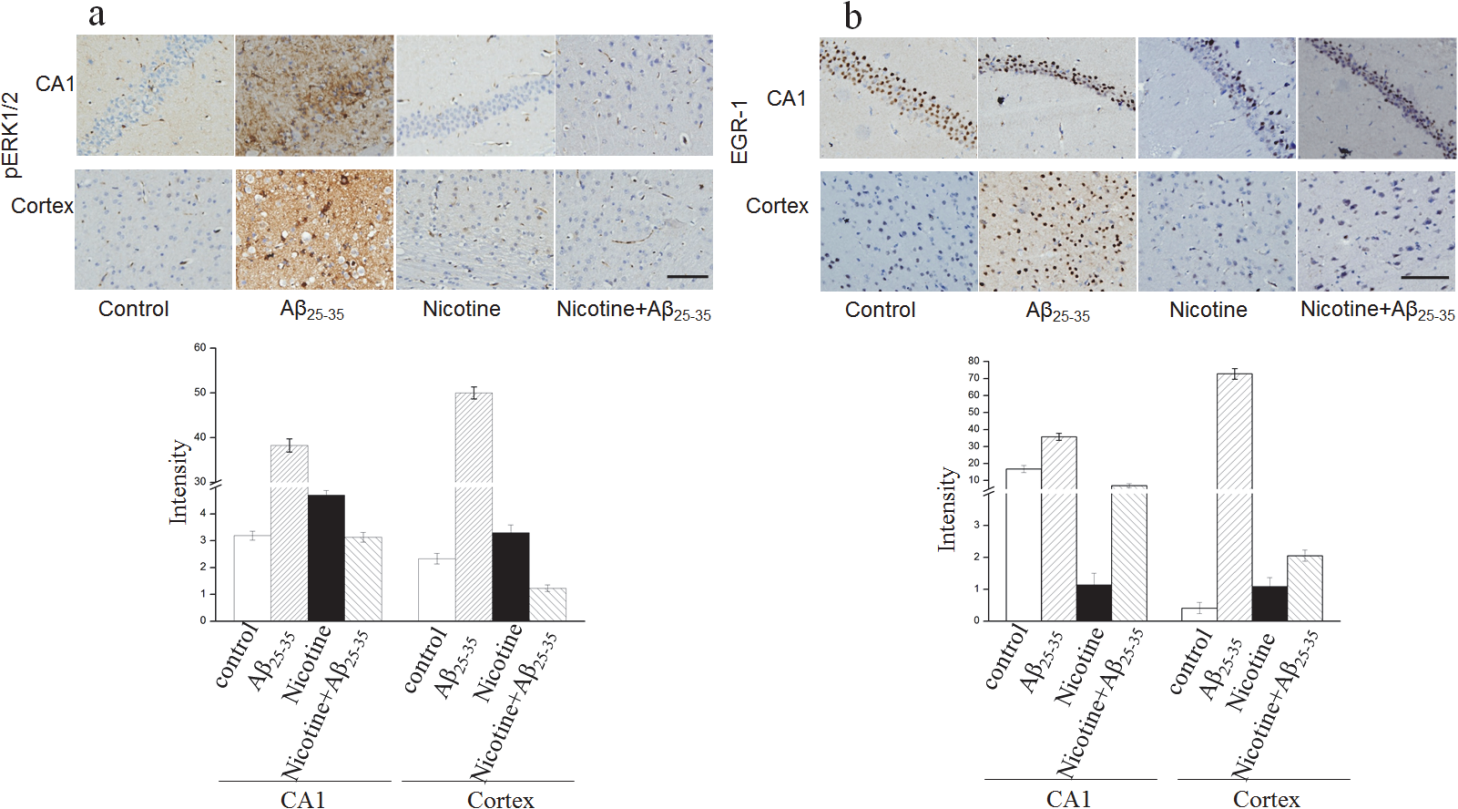
Nicotine antagonizes Aβ_25–35_-induced pERK1/2 and EGR-1 activation in the hippocampus. C57/BL/L6 mice were injected with Aβ_25–35_ or control sodium chloride into hippocampus by stereotaxis injection, the mice were then injected 0.15 mg/kg nicotine using a subcutaneous injection twice a day for 2 weeks. Identical doses of sodium chloride were administered as control. After treatment, the mice were sacrificed and the brain were removed for immunostaining for pERK1/2 and EGR-1. The representive figures of immunostaining activity of pERK1/2 and EGR-1 in CA1 regoin were presented in panel a and b respectivily; the quanfication of pERK1/2 and EGR-1 immunoactivity were shown under the figures.

The in vitro data provided the hint that nicotine could protect the amyloid neuronal toxicity by regulating EGR-1. We next explore this effect in vivo. As mentioned above in the methods, after treatment, all the mice in four groups were sacrificed by anesthesia. The mice brains were collected for cryosection, the expression of EGR-1 and pERK1/2 were immunostained in CA1 area of hippocampus and cortex. As shown in Fig. 6A, Aβ_25–35_ loading increased the ERK1/2 phosphorylation in CA1, while nicotine was an antagonist to Aβ_25–35_-induced the level of pERK1/2. The immunostaining activity of EGR-1 was also increased by injection Aβ_25–35_ in CA1 area and cortex. After the mice receiving nicotine treatment, the up-regulation of EGR-1 in AD mice model was attenuated (Fig. 6B). The software of Image-pro plus 6.0 was used to analyze the immunoactivity of EGF-1 and pERK1/2, and the quantifications were also presented in Fig. 6 respectively. This data suggested that extracellular signal-regulated kinase was an important signaling molecule in synaptic plasticity and memory [28], indicating that signal transmission in hippocampus was essential for the activation of ERK1/2.

## Discussion

Cigarette smoking has been suggested as a risk factor for chronic airway obstruction and lung cancer [29, 30]. Nicotine is the major addictive agent in tobacco. Interestingly, the regular doses of nicotine may be beneficial for human health, especially for the treatment of AD. A variety of studies have shown that acute treatment with nicotine or nicotinic agonists can improve working memory function in rats [7, 8, 23]. Additionally, chronic nicotine treatment prevented Aβ-induced memory impairment [8]. Nicotine was also reported to enhance learning and memory by activating hippocampal Jun-N terminal kinase pathway [31].

Aβ is the major constituent of the senile plaques in AD. It is produced by proteolytic processing of amyloid-β protein precursor (APP), a transmembrance glycoprotein expressed during normal cellular metabolism [32]. Neuronal apoptosis has been implicated in the pathogenesis of various neurodegenerative disorders including AD [33]. Several studies have indicated that Aβ induces apoptosis and neuronal cell death by increasing the expression of iNOS, which leads to the peroxidation of membrane lipids and oxidative stress [34]. According to the previous literature, Aβ-associated free radical damage to neurons is a fundamental process in connection with AD [34]. In this study, treatment of SH-SY5Y cells with Aβ_25–35_ clearly decreased cellular MTT reduction, thereby providing direct evidence for cellular damage induced by Aβ. Nicotine can protect SH-SY5Y cells from Aβ_25–35_-induced cytotoxicity (Fig. 4C). Aβ_25–35_ can increase SH-SY5Y apoptosis compared to control, and nicotine can partly attenuate this Aβ_25–35_-induced cytotoxicity (Fig. 4A and 4B). The ERK pathway plays an important role in the processes related to synaptic plasticity, including learning and memory. *In vivo*, immunohistochemistry indicated that Aβ_25–35_ can up-regulate ERK phosphorylation and activated Egr-1, while nicotine blocked this effect which was induced by Aβ_25–35_ (Fig. 6). Therefore, it is conceivable that the induction of EGR-1 may direct the transcription of downstream genes that are degenerative processes associated with AD. However, additional studies are necessary to confirm the underlying mechanism.

In this work, it was suggested that nicotine activated EGR-1 expression in the human neuroblastoma SH-SY5Y cell line via the stimulation of the MAPK/ERK signaling pathway and the α7 subtype of nAchR. SH-SY5Y cells whose ancestral cell is SK-N-SH were directly subcloned from SH-SY5[35]. The SH-SY5Y cell line expresses α3, α5, α7, β2, and β4 nAChR subunits that assemble to form various α3-nAChR subtypes or homomeric α7 nAChR [36, 37]. The intermediate steps coupling α7 nAChR activation and Ca^2+^ entry to the activation of MEK are likely to depend on stimulus-specific factor [24, 38]. When α7 nAchR was blocked (Fig. 3A, 3B), nicotine-induced ERK1/2 phosphorylation and EGR-1 expression were reduced compared to control (Fig. 3C). The results presented here provide evidence for a functional role for α7 nAChR on SH-SY5Y cells, which is capable of activating the ERK1/2 signaling cascade. To date, considerable interest has been focused on nAChRs in connection with the treatment of AD due to the role it plays in neurotransmitter release, cell survival, cognitive enhancement, synaptic plasticity and neuroprotection [26, 38]. These results suggest that the effects of nicotine exposure may be due to its effects on gene expression initiated by interactions with nAChR. The regulation by the MEK-ERK1/2 signaling pathway after stimulation of the α7 nAChR constitutes a novel signaling cascade in SH-SY5Y cells.

The *egr-1* gene has a serum responsive element (SRE) located within its promoter regions [39]. EGR-1 can be activated by the ERK pathway [22]. We found that the presence of U0126, an ERK pathway inhibitor, effectively weakened the signals of EGR-1, which was triggered by nicotine (Figs. 1, 2C). CyclinD1, as the targeted gene of Wnt/β-catenin signaling pathway, plays an important function in AD [40]. The early growth response protein may bind to a *cis*-regulatory region spanning nucleotides −144 to −104 of the CyclinD1 promoter [41]. The relation between CyclinD1 and EGR-1 in cells treated with 10^–6^ M nicotine for 45 min was revealed (Fig. 5A). When the EGR-1 was blocked, CyclinD1 and its phosphorylation were up-regulated (Fig. 5B, 5C). Therefore, the hypotheses regarding EGR-1 over-expression in this cell line was confirmed (Fig. 5D).

In conclusion, we demonstrated that nicotine activated the EGR-1 via ERK1/2 and the α7 nicotinic acetylcholine receptor in SH-SY5Y cells. Nicotine can protect SH-SY5Y from Aβ-induced cytotoxicity via up-regulating CyclinD1. The results suggested that nicotine could attenuate the effect of Aβ, and it may be beneficial for the prevention of AD. We elucidated the partial mechanisms that underlie the activation of EGR-1 by nicotine, and provide theoretical knowledge for retarding the proceeding of AD.
